# P-1852. Evaluation of Outpatient Parenteral Antimicrobial Therapy and Antimicrobial Stewardship Program Practices at a Veterans Affairs Medical Center

**DOI:** 10.1093/ofid/ofaf695.2021

**Published:** 2026-01-11

**Authors:** Alley Minton, Bailey Guest, Galina Wang, Cassidy Prewitt, Christopher Schrank

**Affiliations:** Salisbury VA Health Care System, Salisbury, North Carolina; Salisbury VA Health Care System, Salisbury, North Carolina; Salisbury VA Health Care System, Salisbury, North Carolina; Salisbury VA Health Care System, Salisbury, North Carolina; Wake Forest School of Medicine, Winston-Salem, North Carolina

## Abstract

**Background:**

Outpatient parenteral antimicrobial therapy (OPAT) refers to the delivery of parenteral antimicrobial treatment in at least two doses on different days without hospitalization required. Implemented antimicrobial stewardship programs (ASP) have reported significant reductions in unnecessary antibiotic prescriptions and improved patient outcomes. At the Salisbury Veterans Affairs Health Care System (SVAHCS), the OPAT program enrolls approximately 150 Veterans annually to which 86% of OPAT requests originate outside of VA care. The purpose of this study was to evaluate the efficacy and safety of OPAT along with ASP practices at SVAHCS.

**Figure:**
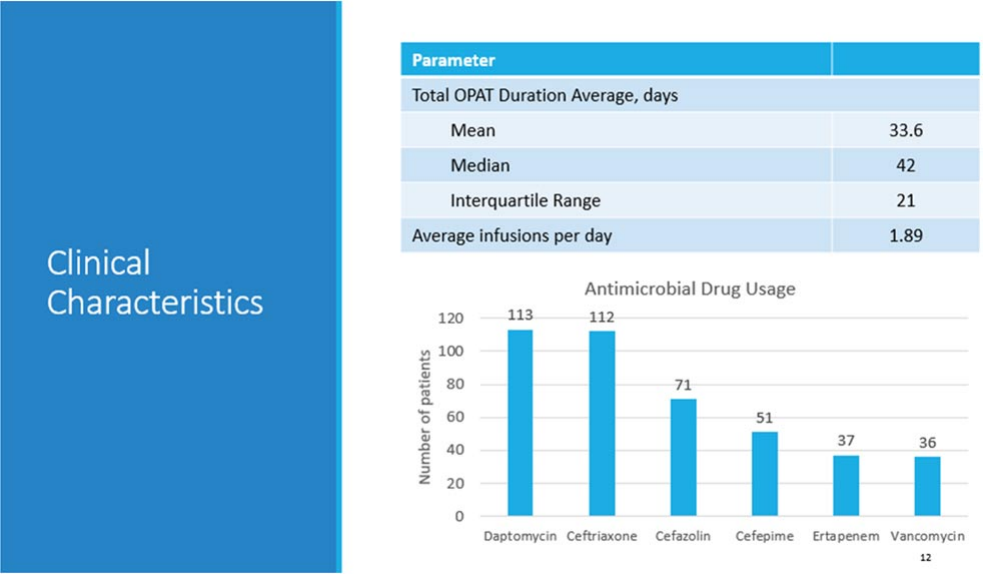
Clinical Characteristics and Most Common OPAT Antimicrobials

**Figure:**
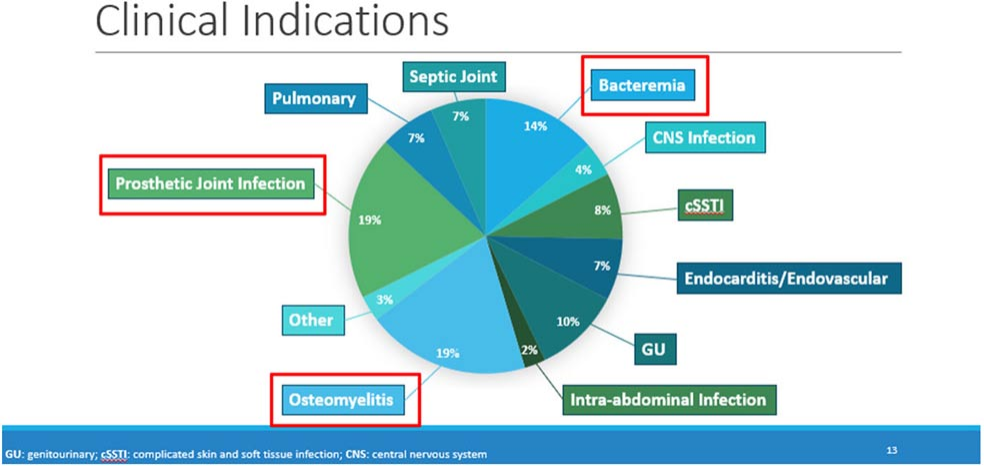
OPAT Indications

**Methods:**

This quality improvement project was conducted as a retrospective cohort study on eligible Veterans on OPAT between January 2022 through December 2024. The main objective was to assess the efficacy of OPAT. The primary outcome was readmission rates to a hospital while receiving OPAT due to complications, adverse events, treatment failure or for reasons unrelated to infection or OPAT regimen. Secondary objectives included evaluating safety, 30-day mortality post-OPAT, 30-day readmissions post-OPAT, rate of OPAT completion, the reduction of hospital bed days of care (BDOC) and intravenous (IV) line days avoided, ASP interventions and potential cost savings.

**Figure:**
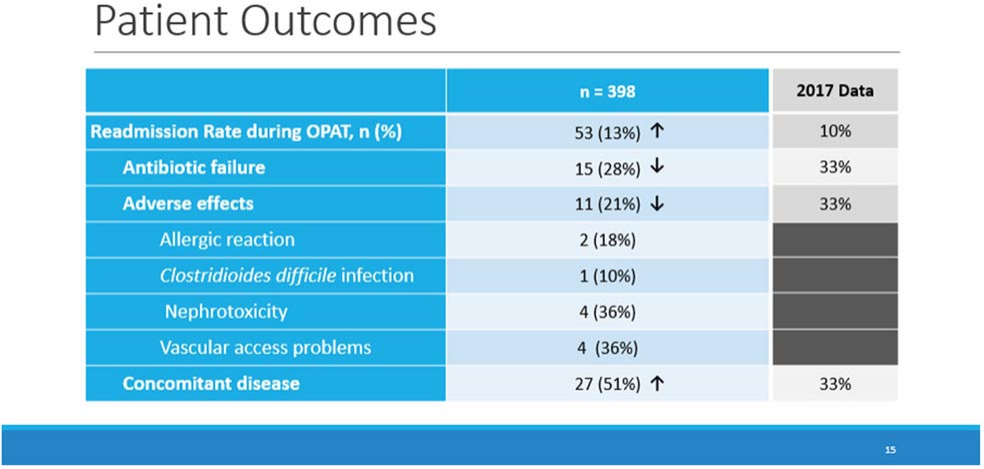
PAT Readmissions during OPAT and Associated Reasons

**Figure:**
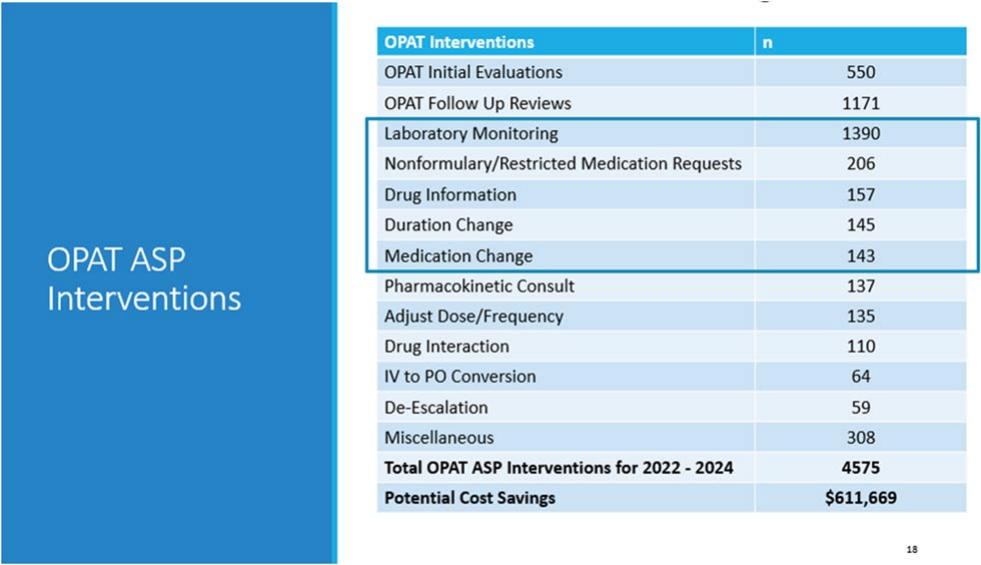
OPAT ASP Interventions and Potential Cost Savings

**Results:**

A total of 398 Veterans were included in this study with 53 (13%) readmissions during OPAT. Of those readmissions, 15 (28%) were secondary to antibiotic failure, 11 (21%) were due to adverse effects and 27 (51%) for concomitant diseases. There were five deaths which equates to a post-OPAT 30-day mortality rate of 1%. Nine readmissions (2%) occurred within 30 days post-OPAT with 2 (22%) due to antibiotic failure, 1 (12%) secondary to adverse effect and 6 (66%) for concomitant diseases. The OPAT completion rate was 97%. A mean of 3,337 BDOC and 134 IV line days were avoided annually. A total of 4,575 ASP interventions associated to OPAT were documented with potential cost savings of $611,669.

**Conclusion:**

The OPAT program and ASP at SVAHCS effectively manage Veterans with low 30-day mortality and adverse effect rates, high OPAT completion rate and comparable readmission rates to current literature while diligently implementing ASP interventions with associated potential cost savings.

**Disclosures:**

All Authors: No reported disclosures

